# UVA, UVB Light, and Methyl Jasmonate, Alone or Combined, Redirect the Biosynthesis of Glucosinolates, Phenolics, Carotenoids, and Chlorophylls in Broccoli Sprouts

**DOI:** 10.3390/ijms18112330

**Published:** 2017-11-04

**Authors:** Melissa Moreira-Rodríguez, Vimal Nair, Jorge Benavides, Luis Cisneros-Zevallos, Daniel A. Jacobo-Velázquez

**Affiliations:** 1 Tecnológico de Monterrey, Escuela de Ingeniería y Ciencias, Centro de Biotecnología FEMSA, Av. Eugenio Garza Sada 2501 Sur, C.P. 64849 Monterrey, N.L., México; a00808666@itesm.mx (M.M.-R.); jorben@itesm.mx (J.B.); 2 Department of Horticultural Sciences, Texas A&M University, College Station, TX 77843-2133, USA; vimaln@tamu.edu (V.N.); lcisnero@tamu.edu (L.C.-Z.)

**Keywords:** UVA UVB light, UV radiation, methyl jasmonate, abiotic stress, glucosinolate profile, phenolic profile, carotenoid profile, chlorophyll profile, broccoli sprouts

## Abstract

Broccoli sprouts contain health-promoting phytochemicals that can be enhanced by applying ultraviolet light (UV) or phytohormones. The separate and combined effects of methyl jasmonate (MJ), UVA, or UVB lights on glucosinolate, phenolic, carotenoid, and chlorophyll profiles were assessed in broccoli sprouts. Seven-day-old broccoli sprouts were exposed to UVA (9.47 W/m^2^) or UVB (7.16 W/m^2^) radiation for 120 min alone or in combination with a 25 µM MJ solution, also applied to sprouts without UV supplementation. UVA + MJ and UVB + MJ treatments increased the total glucosinolate content by ~154% and ~148%, respectively. MJ induced the biosynthesis of indole glucosinolates, especially neoglucobrassicin (~538%), showing a synergistic effect with UVA stress. UVB increased the content of aliphatic and indole glucosinolates, such as glucoraphanin (~78%) and 4-methoxy-glucobrassicin (~177%). UVA increased several phenolics such as gallic acid (~57%) and a kaempferol glucoside (~25.4%). MJ treatment decreased most phenolic levels but greatly induced accumulation of 5-sinapoylquinic acid (~239%). MJ treatments also reduced carotenoid and chlorophyll content, while UVA increased lutein (~23%), chlorophyll *b* (~31%), neoxanthin (~34%), and chlorophyll *a* (~67%). Results indicated that UV- and/or MJ-treated broccoli sprouts redirect the carbon flux to the biosynthesis of specific glucosinolates, phenolics, carotenoids, and chlorophylls depending on the type of stress applied.

## 1. Introduction

Broccoli sprouts have gained recognition as nutraceutical foods due to their high content of health-promoting phytochemicals including phenolic compounds, carotenoids, chlorophylls, and glucosinolates, with concentrations several times greater than the adult plant [1,2]. These defense-related secondary metabolites have been positively linked to several biological properties such as anti-carcinogenic, anti-inflammatory, and antioxidant activity [2]. In this context, broccoli sprouts are considered a valuable phytochemical-rich source of functional foods [3].

Glucosinolates are sulfur-rich secondary metabolites derived from amino acids that can be further hydrolyzed by the endogenous enzyme myrosinase (β-thioglucosidase glucohydrolase, EC 3.2.3.1) into bioactive compounds, e.g., isothiocyanates [4]. In human health, glucosinolates and their hydrolysis products act as indirect antioxidants by modulating the activity of xenobiotic metabolizing (phase I and II) enzymes that trigger long-lasting antioxidant activity, reducing the oxidative stress status that leads to chronic degeneration [5]. They also exhibit cholesterol-lowering, anti-mutagenic, anti-carcinogenic, and anti-inflammatory activity, being associated with reduced risks of cancer of the lungs, stomach, breasts, prostate, pancreas, skin, colon, and rectum [5,6,7].

Phenolic compounds are secondary metabolites produced via the shikimate and phenylpropanoid pathways [8]. Hydroxycinnamic acids and flavonoid glycosides are among the main phenolic compounds found in broccoli [9,10,11]. These compounds possess potent antioxidant activity [3,12] and may play roles in the prevention of diabetes, cardiovascular and neurodegenerative diseases [13,14,15].

Carotenoids are pigments of isoprenoid origin synthesized as secondary metabolites by all photosynthetic organisms such as plants, as they play essential biological roles in light energy collection and protecting the photosynthetic apparatus from photo-oxidative damage [16]. β-carotene and the xanthophylls lutein, zeaxanthin, violaxanthin, neoxanthin, and β-cryptoxanthin are among the major carotenoids found in broccoli [17,18]. Carotenoids show antioxidant and immunomodulation activities, which may prevent degenerative diseases, such as cardiovascular diseases, UV-induced skin damage, cataracts and macular degeneration, diabetes, and several types of cancer, especially prostate and digestive-tract tumors [16,19].

Chlorophylls are another group of light-absorbing pigments present in broccoli sprouts. They are primary metabolites that contain a tetrapyrrole ring (porphyrin) structure [20]. Dietary chlorophyll from fresh fruits and vegetables is predominantly composed of lipophilic derivatives including chlorophyll *a* and *b*, and the biological activities attributed to these derivatives include wound healing; internal deodorization; control of calcium oxalate crystals; anti-inflammatory, antioxidant, and anti-mutagenic activity; mutagen trapping; modulation of xenobiotic metabolism; and induction of apoptosis [21].

The biosynthesis of the abovementioned secondary metabolites is related to the plant endogenous-defense mechanisms, being induced in response to biotic and abiotic stresses [e.g., wounding, ultraviolet (UV) radiation, and exogenous phytohormones], acting as natural compounds to protect the plant [22]. Thus, the application of abiotic stresses can be used as a strategy to accumulate high levels of phytochemicals with potential industrial applications [23].

UV stress causes several physiological and metabolic responses in plants including the production of antioxidant systems, action of reparative enzymes such as DNA photolyases, expression of genes involved in both UV protection and repair, and the accumulation of UV-absorbing (e.g., phenolic compounds and carotenoids) and defense-related (e.g., glucosinolates) phytochemicals [24]. These responses are likely to involve specific UV photoreceptors and signal transduction processes, which lead to the regulation of gene transcription [25]. In this context, UV light, particularly in the UVB (280–320 nm) range, has been used in broccoli sprouts as a tool to induce the biosynthesis and accumulation of flavonoids and glucosinolates [26]. Moreira-Rodríguez et al. [27] recently demonstrated that both UVB and UVA (320–400 nm) lights can positively alter the phenolic and glucosinolate profiles of broccoli sprouts and reported how these profiles can be tailored by controlling the UV light dose and harvesting time.

Elicitor phytohormones, such as methyl jasmonate (MJ), are another class of abiotic stressors that trigger cascades of physiological responses that affect plant morphology and result in the synthesis and accumulation of secondary metabolites [28]. Thus, phytohormones can be artificially applied to plants as an approach to enhance their phytochemical content. In broccoli sprouts, Pérez-Balibrea et al. [29] found that indole glucosinolates responded to the induction with MJ (25 μmol/L) by increasing their concentration by 33%; Barrientos Carvacho et al. [30] reported that MJ (10–90 μmol/L) decreased total polyphenol content in comparison with control broccoli sprouts, but stimulated individual phenolics such as luteoline (not detected in controls).

Given the lack of studies regarding the effect of phytohormones such as MJ on the content and profile of carotenoids and chlorophylls, as well as the combined effect of phytohormones and UV stress on the phytochemical profiles in broccoli sprouts, the objective of the present study was to investigate the effect of the exogenous elicitor MJ alone and in conjunction with UVA or UVB radiation on the accumulation glucosinolates, phenolic compounds, carotenoids, and chlorophylls in broccoli sprouts.

## 2. Results and Discussion

### 2.1. Effect of UVA or UVB Light and Methyl Jasmonate on the Accumulation of Glucosinolates

**Figure 1:**
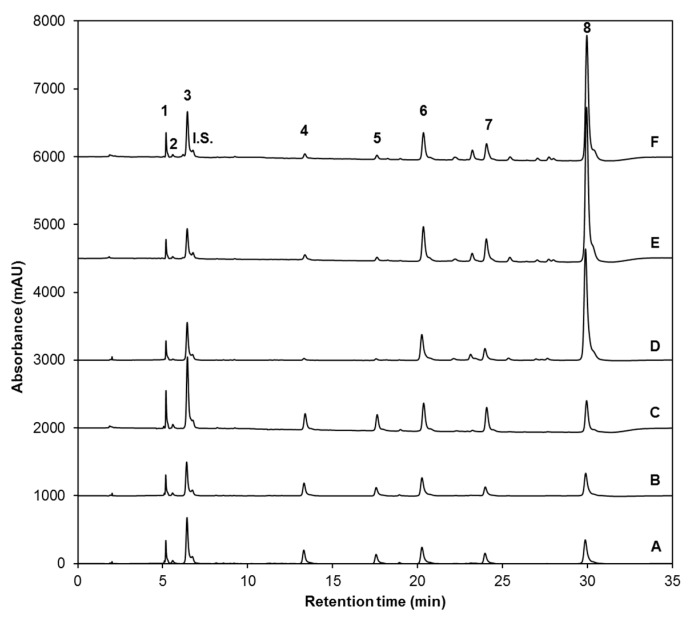
High-Performance Liquid Chromatography with Diode-Array Detection (HPLC-DAD) chromatograms (shown at 227 nm) of identified desulfoglucosinolates (dsg) from ethanol/water (70:30, *v*/*v*) extracts of: (**A**) untreated control eight-day-old broccoli sprouts, broccoli sprouts treated with (**B**) UVA or (**C**) UVB radiation at the 7th day after sowing and harvested 24 h after the UV treatment, (**D**) 8-day-old broccoli sprouts treated with methyl jasmonate (25 µM) every 12 h from sowing until harvest, and broccoli sprouts treated with both methyl jasmonate (25 µM, every 12 h for eight days) and (**E**) UVA or (**F**) UVB radiation at the 7th day after sowing and harvested 24 h after the UV treatment. Peak assignment is shown in Table 1. Glucoiberin-dsg (1); Progoitrin-dsg (2); Glucoraphanin-dsg (3); 4-hydroxy-glucobrassicin-dsg (4); Glucoerucin-dsg (5); Glucobrassicin-dsg (6); 4-methoxy-glucobrassicin-dsg (7); Neoglucobrassicin-dsg (8); Internal standard, sinigrin (I.S.).

Eight glucosinolates were identified in broccoli sprouts in both control and treatment samples (Figure 1 and Table 1). The glucosinolate profile includes four aliphatic glucosinolates: glucoiberin (GIB, compound **1**); progoitrin (PRO, compound **2**); glucoraphanin (GRA, compound **3**); and glucoerucin (GER, compound **5**); and four indolyl glucosinolates: 4-hydroxy-glucobrassicin (4-HGBS, compound **4**); glucobrassicin (GBS, compound **6**); 4-methoxy-glucobrassicin (4-MGBS, compound **7**); and neoglucobrassicin (NGBS, compound **8**). GRA was the glucosinolate found in greater proportion in untreated sprouts (Figure 1A) and sprouts treated with UVA (9.47 W/m^2^ for 120 min) (Figure 1B) or UVB (7.16 W/m^2^ for 120 min) radiation alone (Figure 1C). This agrees with previous literature reports about glucosinolate profiles in broccoli sprouts [26,29]. However, in all 25 µM MJ-treated sprouts (alone or in combination with UVA or UVB radiation), NGBS was the predominant glucosinolate (Figure 1D–F).

The individual and total concentrations of glucosinolates (identified and quantified as desulfoglucosinolates) in broccoli sprouts subjected to MJ, UVA radiation, UVB radiation, or a combination of these stresses are presented in Table 2.

MJ treatment alone significantly increased (*p* < 0.05) the content of total glucosinolates by 97.5%, compared to non-treated sprouts. However, the highest increment in total glucosinolates was observed in both UVA + MJ (153.6% increase) and UVB + MJ (147.9% increase) treatments. These findings agree with previous reports that indicate that the exogenous application of MJ may induce the expression of genes related with the biosynthesis of glucosinolates in *Arabidopsis thaliana* [31]. Moreover, the pre-harvest application of 250 µM MJ has been shown to be effective for inducing the accumulation of total glucosinolates in broccoli florets and sprouts [29,32]. UVB treatment alone also increased total glucosinolate content in a significant manner (by 46.8%), when compared to controls, while UVA treatment alone was not sufficient to induce an overall increment (Figure 1 and Table 2).

Upon analysis of individual glucosinolates, it can be noted that increments induced by MJ (alone or combined with UV radiation) on total glucosinolate content are mainly attributed to the MJ-induced accumulation of the indole glucosinolate NGBS (Figure 1D–F). NGBS was enhanced by 337.7% in broccoli sprouts treated with MJ alone (Table 2). Similarly, Wiesner et al. [33] reported a 38-fold increase in NGBS, in comparison with non-treated samples of sprouts and leaves of pak choi treated with 200 µM MJ. Moreover, NGBS was synergistically enhanced by 538.4% and 514.5% when MJ was applied in conjunction with UVA and UVB, respectively. No significance difference was detected between samples treated with UVA and UVB in combination with MJ (Table 2). Thus, when combined MJ and UV, a redirection in glucosinolate biosynthesis towards NGBS was observed, making this combined stress a synergistic effect in detrimental to the other individual glucosinolate compounds (Table 2).

Likewise, MJ treatment induced the biosynthesis of two other indole glucosinolates, one of them being GBS, the parental glucosinolate in the indole biosynthetic pathway. In particular, UVA + MJ treatment increased the content of GBS by 72.4%, followed by UVB + MJ (47.7%) and MJ (46.77%) (Table 2). 4-MGBS is the other indole glucosinolate positively affected by MJ in a similar pattern as NGBS, since UVA + MJ, UVB + MJ, and MJ caused increases in this glucosinolate by 131.1%, 104.6% and 40%, respectively. However, it is worth mentioning that UVB treatment alone was responsible for the highest accumulation of 4-MGBS (increased by 177.5%) (Table 2). The remaining indole glucosinolate identified in the samples, 4-HGBS was negatively affected by MJ treatment (Table 2). MJ alone decrease its content by 91.1%, followed by UVB + MJ (decreased by 75.8%) and UVA + MJ (decreased by 69.8%) treatments.

These results agree with Mikkelsen et al. [31] and Skirycz et al. [34], who reported that MJ treatment induces the accumulation of indolyl glucosinolates, as it can induce transcription factors involved in their biosynthesis (i.e., MYB51, MYB34, and OBP2). Additionally, in the indole glucosinolate biosynthetic pathway, GBS is synthesized by sulfotransferases 16 and 18 (SOT16 and SOT18), then GBS is converted into the intermediates 1-hydroxy-glucobrassicin (1-HGBS) or 4-HGBS by hydroxylation reactions catalyzed by the enzymes CYP81F1-3 and CYP81F4, respectively. These glucosinolates are further converted to NGBS and 4-MGBS, respectively, by methylation reactions catalyzed by a specific class of plant family 2-*O*-methyltransferases (IGMT1 and IGMT2 for both intermediates and IGMT5 for 1-HGBS only) [33,35,36]. Therefore, the observations presented herein suggest that in broccoli sprouts, the methylation of GBS is favored by pre-harvest application of MJ treatment and that such an effect is synergistically enhanced for NGBS by the addition of UV treatment. Moreover, Pfalz et al. [36] recently proposed that biosynthesis of NGBS from GBS in *A. thaliana* involves formation of its highly unstable intermediate, 1-HGBS, and that IGMT5 is responsible for the conversion of 1-HGBS to NGBS. Thus, it is likely that the absence of 1-HGBS in broccoli sprouts confirms that biosynthesis favors the production of NGBS; and that the family of IGMTs, especially IGMT5, might be synergistically activated by the combination of UV and MJ stresses. This partially explains why NGBS is accumulated at significantly higher levels than its counterpart, 4-MGBS, whose formation does not depend on IGMT5 and whose intermediate (4-HGBS) is present in the samples of broccoli sprouts and is indeed decreased by MJ treatment as it is further converted into 4-MGBS (Table 2).

Despite the induction of indole glucosinolate biosynthesis, MJ treatments did not increase the concentration of any of the four aliphatic glucosinolates identified. GIB and GRA remained unaffected after MJ treatments; PRO was significantly affected (decreased by 46.8%) by MJ alone but was not affected when treated with MJ + UV. Content of GER decreased after all MJ treatments in a similar manner to 4-HGBS: MJ alone caused a decrease of 93.6%, followed by UVA + MJ (decreased by 79.6%) and UVB + MJ (decreased by 77.5%) treatments. In agreement, Pérez-Balibrea [29] reported decreases in aliphatic glucosinolate concentrations in broccoli sprouts treated with 10, 25, 50, and 100 µM MJ solutions; the negative effect of MJ was greater with increased concentrations of MJ. This might be related to the incapacity of MJ to induce the activity of aliphatic transcription factors other than MYB28 [37]. Thus, these observations further confirm the likelihood that MJ selectively induces activation of the biosynthetic machinery on the indole branch of the glucosinolate pathway, rather than the aliphatic branch, thus explaining why (a) increments in individual aliphatic glucosinolates were not observed when MJ was exogenously applied to broccoli sprouts and (b) contents of individual aliphatic glucosinolates were not as low when UVA or UVB stress was combined with MJ, as compared to MJ treatment alone.

Regarding the independent effects of UVA or UVB radiation (Figure 1B,C, respectively), UVB alone (Figure 1C and Table 2) caused significant increases in all but two glucosinolates; the aliphatic GIB, GRA, and GER increased only after UVB treatment by 84%, 78.1%, and 62.6%, respectively, while the indole GBS, 4-HGBS, and 4-MGBS increased by 30%, 31.8%, and 177.5%, respectively, as compared with non-treated sprouts. These results agree with a previous report where UVB radiation induced an accumulation of glucosinolates, mainly GRA and 4-MGBS in 12-day-old broccoli sprouts 24 h after exposure, triggered by an upregulation in transcript levels of genes related to secondary metabolite biosynthesis pathways, and stress response in the broccoli sprouts [26]. Likewise, in *A. thaliana* UVB radiation elicited an increase of GRA and 4-MGBS [38].

Additionally, it has been stated that plant responses to UVB partially overlap those of defense signaling induced by wounding, insects, and pathogens [39], which are regulated by MJ [40,41]. In broccoli sprouts, UVB radiation induced the production of reactive oxygen species (ROS), which in turn caused the up-regulation of genes associated with salicylate (SA) and jasmonic acid (JA; precursor of MJ) signaling, with pathogen attack and/or wounding, such as *PR-4* and *BG3*, leading to the production of glucosinolates as a plant defense mechanism [26]. Likewise, Villarreal-García et al. [11] reported a synergistic effect between wounding and MJ stresses (250 ppm), inducing a 286% increase in NGBS. Hence, the synergistic effect observed in the present study when MJ and UVB stresses were applied to broccoli sprouts likely involves an upregulation of the JA-/SA-/ROS-related signaling pathways by both UVB and MJ stress.

On the other hand, all individual glucosinolates remained unaltered after UVA radiation (only GER was significantly decreased by 27.6%), compared with controls (Figure 1B and Table 2). However, a combination of UVA radiation with MJ treatment accounted for the highest total glucosinolate content (153.6% increase). The latter was mainly due to the induction of the indole glucosinolates GBS (by 538.4%), 4-MGBS (by 131.1%), and NGBS (by 72.4%) when both stresses were applied (Figure 1E and Table 2). Since little is known about the mechanisms governing the effects of UVA on glucosinolate accumulation, it can only be presumed that UVA radiation induces signal-transduction responses (similar to those exerted by UVB radiation) only when MJ stress is present, and that these signaling responses may couple with those induced by MJ to favor the accumulation of indole glucosinolates.

### 2.2. Effect of UVA or UVB Light and Methyl Jasmonate on the Accumulation of Phenolic Compounds

**Figure 2:**
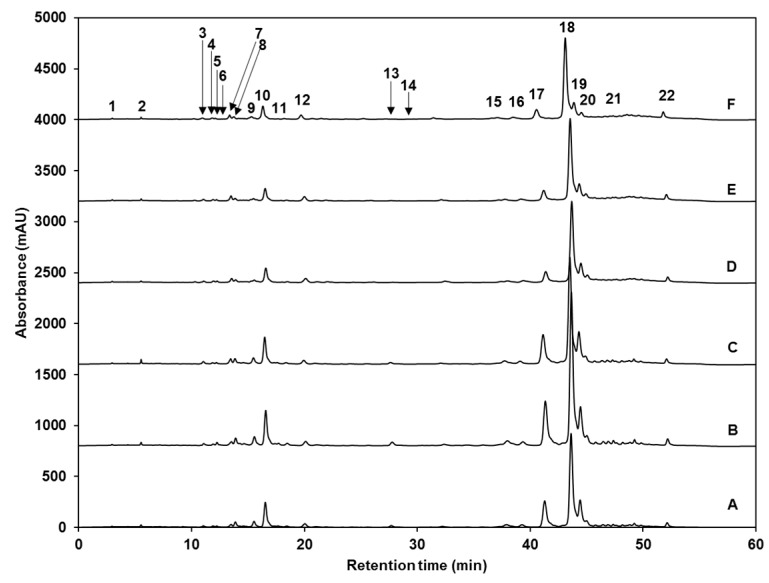
HPLC-DAD chromatograms (shown at 320 nm) of identified phenolic compounds from ethanol/water (70:30, *v/v*) extracts of: (**A**) untreated control eight-day-old broccoli sprouts, broccoli sprouts treated with (**B**) UVA or (**C**) UVB radiation at the 7th day after sowing and harvested 24 h after the UV treatment; (**D**) eight-day-old broccoli sprouts treated with methyl jasmonate (25 µM) every 12 h from sowing until harvest, and broccoli sprouts treated with both methyl jasmonate (25 µM, every 12 h for eight days) and (**E**) UVA or (**F**) UVB radiation at the 7th day after sowing and harvested 24 h after the UV treatment. Peak assignment is indicated in Table 3. Gallic acid hexoside I (1); gallotannic acid (2); *p*-hydroxybenzoic acid (3); gallic acid hexoside II (4); 4-*O*-caffeoylquinic acid (5); digalloyl hexoside (6); 3-*O*-hexoside kaempferol (7); gallic acid derivative (8); 1-*O*-sinapoyl-β-d-glucose (9); sinapoyl malate (10); 1,2-diferulolylgentiobiose (11); 5-sinapoylquinic acid (12); sinapic acid (13); gallic acid (14); kaempferol 3-*O*-sinapoyl-sophoroside 7-*O*-glucoside (15); 1,2-disinapoylgentiobiose (16); 1-sinapoyl-2′-ferulolylgentiobiose (17); 1,2,2′-trisinapoylgentiobiose (18); 1,2-disinapoyl-1′-ferulolylgentiobiose (19); 1,2-disinapoyl-2-ferulolylgentiobiose (20); 1-sinapoyl-2,2′-diferulolylgentiobiose (21); (isomeric) 1,2,2′-trisinapoylgentiobiose (22).

The phenolic content of broccoli sprouts treated with UV light, MJ, and a combination of stresses was also investigated. Twenty-two major phenolic compounds were identified in both control and UV-treated broccoli sprouts (Figure 2 and Table 3). Identified phenolics (listed according to their retention time specified in Table 3) include gallic acid hexoside I (GAH I, **1**) and gallic acid hexoside II (GAH II, **4**); gallotannic acid (GTA, **2**); *p*-hydroxybenzoic acid (*p*-HBA, **3**); 4-*O*-caffeoylquinic acid (4-*O*-CQA, **5**); digalloyl hexoside (diGH, **6**); 3-*O*-hexoside kaempferol (3-*O*-H-K, **7**); gallic acid derivative (GAD, **8**); 1-*O*-sinapoyl-β-d-glucose (1-*O*-S-β-d-g, **9**); sinapoyl malate (**10**); 1,2-diferulolylgentiobiose (1,2-diFG, **11**); 5-sinapoylquinic acid (5-SQA, **12**); sinapic acid (**13**); gallic acid (**14**); kaempferol 3-*O*-sinapoyl-sophoroside 7-*O*-glucoside (K-3-*O*-S-so-7-*O*-g, **15**); 1,2-disinapoylgentiobiose (1,2-diSG, **16**); 1-sinapoyl-2′-ferulolylgentiobiose (1-S-2-FG, **17**); 1,2,2′- trisinapoylgentiobiose and its isomer (1,2,2-triSG, **18, 22**); 1,2-disinapoyl-1′-ferulolylgentiobiose (1,2- diS-1-FG, **19**); 1,2-disinapoyl-2-ferulolylgentiobiose (1,2-diS-2-FG, **20**); and 1-sinapoyl-2,2′-diferulolylgentiobiose (1-S-2,2-diFG, **21**).

Regarding total phenolic content (quantified as the sum of all individual compounds), none of the treatments caused a significant (*p* < 0.05) increase, compared to control sprouts; moreover, all three MJ treatments caused a significant (*p* < 0.05) decrease by ~27, ~25, and ~29%, for MJ, UVA + MJ, and UVB + MJ, respectively (Table 4). The latter does not agree with Pérez-Balibrea et al. [29], who reported that total phenolic concentration increased in seven-day-old broccoli sprouts treated with 25 μM MJ, mainly due to a 25% enhancement in flavonoid content. However, it agrees with a previous report from Barrientos Carvacho et al. [30], in which MJ treatment (10, 50 or 90 μM) applied to broccoli seeds significantly decreased the total phenolic content in the 11-day-old broccoli sprouts, compared with control sprouts.

Changes in phenolic content can be observed when analyzing the concentration of individual phenolics (Table 4). Although scientific efforts to increase phenolic content in plants applying UV stress focus on and suggest mainly the use of UVB light, here UVB alone only increased the levels of GAH II (35.1%) in broccoli sprouts, while UVA radiation alone caused significant (*p* < 0.05) increases in the content of GAH I (10.5%), 1-*O*-S-β-d-g (17.2%), sinapic acid (23.1%), gallic acid (57.2%), K-3-*O*-S-so-7-*O*-g (25.4%), 1-S-2-FG (23.5%), and the second isomer of 1,2,2-triSG (18.9%). The UVA-mediated accumulation in phenolic composition observed herein is likely controlled by the UVA-induced transcript accumulation of genes involved in the phenylpropanoid biosynthetic genes (e.g., *PAL* and *CHS*), the induced activity of PAL enzyme and the UVA absorption through the UVA-specific photoreceptor, cryptochrome 1, which is required for the expression of phenylpropanoid genes [42].

Only one compound, 1,2-diS-2-FG, was affected by both UVA and UVB stresses (decreased by 37% and 56%, respectively), while the other three treatments where MJ was applied did not induce a change in its content. On the contrary, compared to control sprouts, the individual concentration of most phenolic compounds was significantly reduced with the same severity by all treatments involving MJ application, while their concentration remained unaffected (or increased) after UVA or UVB treatments alone. The 12 compounds affected with this pattern were GAH I; GTA; diGH; 3-*O*-H-K; 1-*O*-S-β-d-g; sinapoyl malate; sinapic acid; K-3-*O*-S-so-7-*O*-g; 1,2-diSG; 1-S-2-FG; the majoritarian isomer of 1,2,2-triSG; and 1,2-diS-1-FG (Table 4). These findings agree with the previous work of Villarreal-García et al. [11], in which exogenous application of MJ to broccoli florets repressed the accumulation of specific phenolic compounds, including 1-S-2-FG; 1,2,2-triSG, and 1,2-diS-2-FG, and caused a 19% decrease in the content of 4-*O*-CQA. In the present study, none of the treatments affected the levels of 4-*O*-CQA. Furthermore, Kim et al. [43] recently demonstrated that indole glucosinolate biosynthesis limits phenylpropanoid accumulation in *A. thaliana* due to the high levels of the intermediate indole-3-acetaldoxime, the subsequent metabolite, or the pool of initial amino acids. Thus, the repression of phenolic compounds in MJ-treated broccoli sprouts is likely related to the MJ-induced overproduction of indole glucosinolates, such as NGBS. An alternative hypothesis could be that MJ application induced a large increase in 5-SQA, and thus all other phenolics decreased because of the redirection of the carbon source towards the biosynthesis of this compound.

Moreover, previous reports showed that JA downregulates genes involved in the biosynthesis of phenolic compounds, such as *PAL* and *4-coumarate-CoA ligase* (*4CL*), as well as genes involved in the biosynthesis of lignin, such as the *caffeoyl-CoA 3-O-methyltransferase* (*CCoAOMT*) gene [44]. Therefore, the observed MJ-induced decrease and repression of accumulation of some individual phenolic compounds are likely due to a downregulation of genes involved in the secondary metabolic pathways leading to the biosynthesis of phenolic compounds, and to an increase in the gene regulating the biosynthesis of 5-SQA.

However, MJ and UV light + MJ treatments induced a significant accumulation of a few compounds (Table 4). For instance, MJ alone accumulated gallic acid (by 25.8%); UVA + MJ increased *p*-HBA (by 14.4%) and GAH II (by 41.6%); while UVB + MJ induced the accumulation of the second isomer of 1,2,2-triSG (by 18%). All three MJ treatments increased the levels of 1,2-diFG (by 15–32%) and most notably 5-SQA (increased by 139–239%); in these two cases the elicitor effect of the treatments was observed as MJ *>* UVA + MJ *>* UVB + MJ. Therefore, the results presented herein indicate that MJ is a selective elicitor for the phenylpropanoid pathway in broccoli sprouts, since selectively induces the accumulation of 5-SQA in both UV-treated and non-treated sprouts. The overproduction of 5-SQA might be of commercial and scientific interest as sinapic acid derivatives have drawn recent attention due to their antimicrobial, anti-inflammatory, anti-cancer, and anti-anxiety activity [45].

### 2.3. Effect of UVA or UVB Light and Methyl Jasmonate on the Accumulation of Carotenoids and Chlorophylls

**Figure 3:**
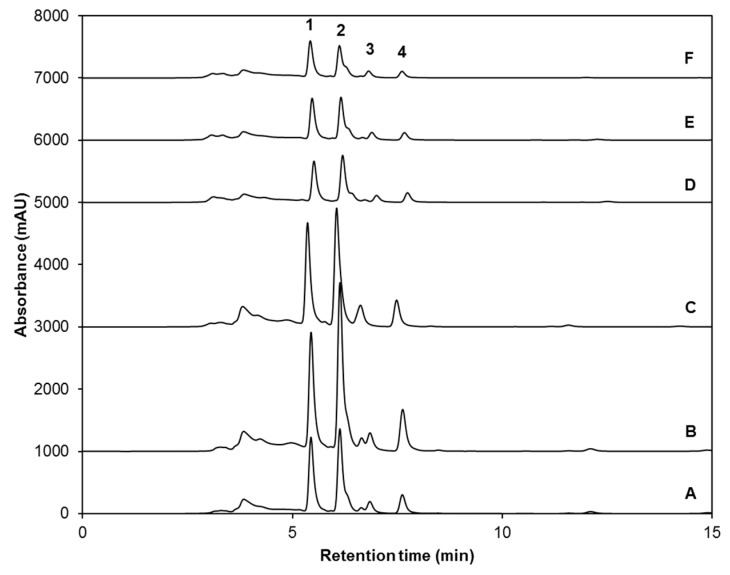
HPLC-DAD chromatograms (shown at 450 nm) of identified carotenoids and chlorophylls from ethanol/water (70:30, *v/v*) extracts of: (**A**) untreated control eight-day-old broccoli sprouts, broccoli sprouts treated with (**B**) UVA or (**C**) UVB radiation at the 7th day after sowing and harvested 24 h after the UV treatment; (**D**) eight-day-old broccoli sprouts treated with methyl jasmonate (25 µM) every 12 h from sowing until harvest, and broccoli sprouts treated with both methyl jasmonate (25 µM, every 12 h for eight days) and (**E**) UVA or (**F**) UVB radiation at the 7th day after sowing and harvested 24 h after the UV treatment. Peak assignment is shown in Table 5. Lutein (1); Chlorophyll *b* (2); Neoxanthin (3); Chlorophyll *a* (4).

The HPLC-DAD carotenoid/chlorophyll chromatogram of control broccoli sprouts and those subjected to UV and/or MJ treatments is shown in Figure 3. Two carotenoids, the xanthophylls lutein and neoxanthin, were identified within the samples, whereas chlorophyll *a* and chlorophyll *b* were also found (Table 5). The carotenoid/chlorophyll chromatogram of broccoli sprouts is similar to those reported in literature for broccoli florets and broccoli sprouts [18,46]. In consistency with Kopsell and Sams [46], chlorophyll *b* was the most abundant photosynthetic pigment found in broccoli sprouts samples, while lutein is usually attributed as the major xanthophyll in broccoli florets [17,18]. Likewise, Kopsell and Sams [46] also agree that neoxanthin is the major light-harvesting xanthophyll present in broccoli sprouts, although they were able to quantify small levels of violaxanthin, antheraxanthin, and zeaxanthin in their 19-day-old sprouts. These differences could be due to a difference in cultivars used as well as the growing conditions and growth stage/age of the plant itself.

Total and individual concentrations of carotenoids and chlorophylls in broccoli sprouts treated with UVA or UVB radiation, MJ or a combination of stresses are presented in Table 6. Compared with untreated broccoli sprouts, the total carotenoid/chlorophyll content was significantly (*p* < 0.05) higher by 36% in UVA-treated sprouts. UVB treatment did not cause a significant increase in total carotenoids + chlorophylls, although it increased the content of individual compounds. Application of 25 µM MJ for 8 days decreased total carotenoid / chlorophyll content in broccoli sprouts by 50.6%, 55.4% and 65% for treatments MJ, UVA + MJ, and UVB + MJ, respectively (Table 6).

Individual carotenoid and chlorophyll compounds were affected in a similar manner. For instance, UVA exposure caused increments in lutein (by 22.4%), chlorophyll *b* (by 30.7%), neoxanthin (by 33.5%), and chlorophyll *a* (by 67%). The accumulation of these compounds after treatment of sprouts with UVA light might be related to the ability of UVA to mediate photosynthetic enhancements via several mechanisms, including (*i*) direct absorption of UVA by chlorophylls and light-harvesting xanthophylls (e.g., lutein and neoxanthin); and (*ii*) absorption by photosynthetic pigments of UVA-induced blue-green fluorescence emitted by phenolic compounds located within the cuticle, or bound in epidermal and vascular tissue cell walls of leaves [42]. Activation of these mechanisms allows UVA light to directly excite and accumulate chlorophyll *a* and its accessory pigments (chlorophyll *b* and light-harvesting xanthophylls) as required [42].

UVB alone also significantly increased lutein (by 16.9%), neoxanthin (by 36.7%) and chlorophyll *a* (by 36.8%), compared to untreated broccoli sprouts (Table 6 and Figure 3B,C). These results are not consistent with a previous report in 12-day-old broccoli sprouts, where no differences in β-carotene (the only carotenoid detected in those broccoli sprouts), chlorophyll *a* or chlorophyll *b* upon 0.042 Wh/m^2^ UVB treatment for 4 h followed by an adaptation time of 24 h [26]. However, Kolterman et al. [52] demonstrated the photooxidative respond of leafy *Brassica juncea* plants to UVB at a low dosage of 0.05 Wh/m^2^ (adaptation time of 20 h), showing a significant increase in carotenoids, mainly β-carotene. Thus, since broccoli sprouts studied herein responded to doses of 7.16 W/m^2^ UVB and 9.47 W/m^2^ UVB for 120 min, it might be assumed that in broccoli sprouts, exposure to high doses of UVB radiation can be used in order to achieve significant accumulation of carotenoids and chlorophylls that contribute to a stress-mediated plant protective mechanism.

Furthermore, the presence of zeaxanthin was originally expected in samples treated with UVA and UVB. Under excessive illumination and radiation, plants activate a mechanism to regulate photosynthesis in which the carotenoid violaxanthin (the neoxanthin precursor) is converted into zeaxanthin, which modulates the photosynthetic apparatus and increases photoprotection at various levels, including dissipation of energy as heat and scavenging of ROS, since it dissipates any singlet oxygen that may form. When light goes back to normal intensities, zeaxanthin is converted back to violaxanthin, closing the so-called xanthophyll cycle [20]. Thus, the absence of zeaxanthin in the UV-treated samples may be attributed to the absence of violaxanthin under normal conditions and, more likely, to the 24 h period of acclimatization that was given to UV-treated broccoli sprouts prior to their harvest, which is supported by the fact that the xanthophyll cycle is activated or deactivated on a minute timescale [20].

On the other hand, MJ alone or combined with UV light caused losses in all individual compounds in the range of 40–50% (Table 6 and Figure 3D–F). This observation is consistent with previous reports where the total content of carotenoids in barley leaf decreased by 60% and 65% after one and three days of MJ treatment, respectively [53], while chlorophyll degradation was accelerated after MJ treatment in apple peel [54] and *A. thaliana* [55], which, according to the authors, might be caused by a MJ-induced stimulation of both ethylene formation and a senescence-like symptom in which the photosynthetic electron transport rate (ETR) and the *F*_v_*/F*_m_ ratio, both indicators of the activity of plant Photosystem II, are decreased.

**Figure 4:**
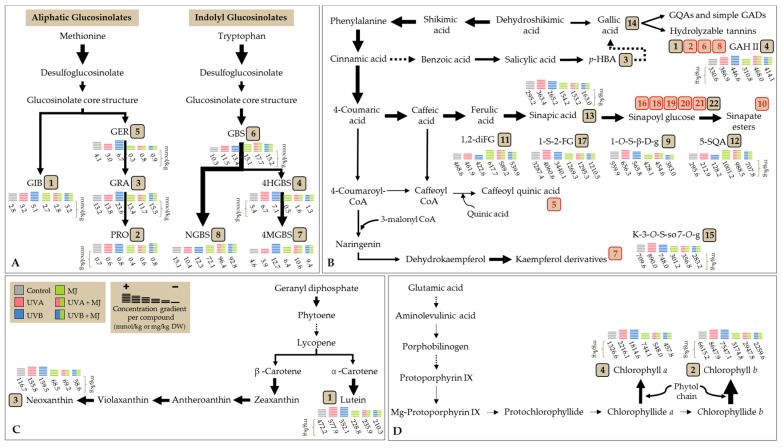
Accumulation of individual (**A**) glucosinolates; (**B**) phenolic compounds; (**C**) carotenoids; and (**D**) chlorophylls in broccoli sprouts treated with UV light and MJ. Identified compounds are located in their corresponding biosynthetic pathway. Dashed arrows represent multiple enzymatic steps. The numbering of compounds corresponds to the peak number assigned in Table 1 for **A**, Table 3 for **B**, and Table 5 for **C** and **D**. Numbers in red correspond to compounds whose concentration decreased or remained unaffected by all treatments compared to its control; and in black, increased. From the latter group, phenolic compounds 4, 9, 11, 12, 15, and 17 (in **B**) were taken as the most representative. Concentrations (in mmol/kg DW or mg/kg DW) correspond to data presented in Table 2 for **A**, Table 4 for **B**, and Table 6 for **C** and **D**. For treatments with MJ (green), 65 mL of a 25 µM MJ solution were exogenously sprayed to broccoli sprouts every 12 h from sowing day until the 8th day after sowing. For treatments with UVA (red) and UVB (blue) lights, broccoli sprouts were exposed for 120 min to doses of 9.47 and 7.16 W/m^2^ respectively, on the 7th day after sowing. Harvest of treated and non-treated sprouts occurred on the 8th day after sowing. The higher the color block, the greater the compound’s accumulation after a given treatment. Abbreviations: Dry weight (DW); Methyl Jasmonate (MJ); Ultraviolet (UV); Glucoiberin (GIB); Progoitrin (PRO); Glucoraphanin (GRA); 4-hydroxy-glucobrassicin (4-HGBS); Glucoerucin (GER); Glucobrassicin (GBS); 4-methoxy-glucobrassicin (4-MGBS), Neoglucobrassicin (NGBS); Galloyl quinic acid (GQA); Gallic acid derivative (GAD); Gallic acid hexoside II (GAH II); *p*-hydroxybenzoic acid (*p*-HBA); 1-*O*-sinapoyl-β-d-glucose (1-*O*-S-β-d-g); 1,2-diferulolylgentiobiose (1,2-diFG); 5-sinapoylquinic acid (5-SQA); Kaempferol 3-*O*-sinapoyl-sophoroside 7-*O*-glucoside (K-3-*O*-S-so-7-*O*-g); 1-sinapoyl-2′-ferulolylgentiobiose (1-S-2-FG).

Finally, a schematic representation of the individual phytochemicals accumulated in broccoli sprouts treated with UVA or UVB light, MJ, and a combination of UV + MJ is shown in Figure 4 to summarize the findings presented herein. Black arrows emphasize the direction of the carbon flux through the glucosinolate, phenolic, carotenoid, and chlorophyll biosynthetic pathways (Figure 4A–D). The diagram serves as a visual tool to select one or more treatments to enhance the content of desired phytochemicals and understand how carbon flux is redirected to the biosynthesis of specific compounds under the type of stresses applied. For instance, the higher blue blocks in Figure 4A,C,D lead to the conclusion that the application of UVB treatment alone may be used to simultaneously accumulate most glucosinolates and all xanthophylls and chlorophylls in broccoli sprouts; whereas UVA + MJ treatment (red with green) may be suitable to produce broccoli sprouts with enhanced content of indolyl glucosinolates and certain phenolics such as 5-SQA (Figure 4).

## 3. Materials and Methods

### 3.1. Chemical and Plant Material

Sulfatase (from *Helix pomatia*), sinigrin hydrate, sephadex A-25, sodium acetate, orthophosphoric acid, sinapic acid, ferulic acid, gallic acid, 3-*O*-caffeoylquinic acid (3-*O*-CQA), lutein, chlorophyll *a* (from *Anacystis nidulans* algae) and chlorophyll *b* (from spinach) were obtained from Sigma-Aldrich Co. (St. Louis, MO, USA) and desulfoglucoraphanin was obtained from Santa Cruz Biotechnology (Dallas, TX, USA). Acetonitrile (HPLC grade) and methanol (HPLC grade) were obtained from Desarrollo de Especialidades Químicas, S.A. de C.V (Monterrey, México), ethanol (HPLC grade) and methyl tert-butyl ether (MTBE, HPLC grade) were obtained from Control Técnico y Representaciones, S.A. de C.V (Monterrey, México). Deionized water (18.2 MΩ·cm resistance) was used in all procedures and was obtained from a Milli-Q Element water purification system (Millipore, Bedford, MA, USA).

Broccoli (*Brassica oleracea* L., var. *italica*, cv. Waltham 29) seeds, Sun Gro Horticulture’s Canadian *Sphagnum* peat moss substrate and Landmark Plastic Corporation’s propagation trays were obtained from IMAISA (Monterrey, México).

### 3.2. Sprouting Method

The sprouting method was adapted from Martínez-Villaluenga et al. [56]. Briefly, broccoli seeds (0.5 g per replication) were sanitized for 15 min in sodium hypochlorite (1.5%, *v/v*), rinsed with Milli-Q water and soaked with aeration overnight in darkness and at room temperature. After pouring off the soaking water, the seeds were spread evenly on standard 200 square cell plug trays (21.38" × 11.05" × 1.75") containing Canadian Sphagnum peat moss previously moistened. Sprouts were grown in a culture room with controlled temperature (25 °C) and a photoperiod regime with cycles of 16 h light and 8 h darkness. Water (control) or a phytohormone solution were atomized every 12 h throughout the experiment.

### 3.3. UV and MJ Treatments

Six trays with broccoli sprouts seeds were prepared for this study, and were assigned for (**A**) Control (no UV or phytohormone application), (**B**) UVA treatment, (**C**) UVB treatment, (**D**) MJ treatment, (**E**) UVA + MJ treatment, and (**F**) UVB + MJ treatment.

MJ treatments (**D**, **E** and **F**) were conducted based on Pérez-Balibrea et al. [29] with slight adjustments. Briefly, MJ was dissolved in 0.2% ethanol to obtain a 25 µM solution and applied every 12 h by exogenous spraying 65 mL of 25 µM MJ solution from sowing day until the end of the experiment (8th day after sowing). Due to the volatility of MJ and to avoid the fact that treatment to one tray may result in application to neighboring trays, the MJ solution was applied using physical separation. Control sprouts (**A**) and sprouts treated with UVA or UVB alone (**B** and **C**) were irrigated with the same frequency using 65 mL of Milli-Q water containing 0.04% ethanol.

On the 7th day after sowing, UV (**B** and **C**) and UV + MJ (**E** and **F**) treatments were carried out in special UVA and UVB chambers based on Moreira-Rodríguez et al. [27] with slight adjustments. Chambers used for treatments **B** and **E** were equipped with two 40 W UVA lamps (Sylvania F40W T12 BL350, Ledvance LLC., Wilmington, MA, USA), while chambers for treatments **C** and **F** consisted of two 40 W UVB lamps (Philips TL 40W/12 RS, Philips, Ljubljana, Slovenia). Trays with broccoli sprouts were placed 30 cm below the irradiation source. All UV treatments consisted of a single exposure for 120 min. The irradiation intensities were determined prior to the experiment as 9.47 and 7.16 W/m^2^ for UVA and UVB, respectively, using a PMA 2200 radiometer equipped with PMA 2110 UVA and PMA 2106 UVB sensors (Solar Light, Glenside, PA, USA) measuring in the spectral range from 320–400 nm and 280–320 nm, respectively.

After UV treatments, trays were returned to culture room and the proper irrigation with water or MJ solution continued for an additional (acclimatization) period of 24 h. Sprouts of all six trays were harvested at the 8th day after sowing, immediately flash-frozen in liquid nitrogen, placed at −80 °C, freeze-dried (Labconco, Kansas City, MO, USA), and then ground to a fine powder. Samples were stored at −80 °C until further analysis.

### 3.4. Phytochemical Analyses

#### 3.4.1. Extraction of Phytochemicals

Extraction of all phytochemicals evaluated (glucosinolates, phenolic compounds, carotenoids and chlorophylls) from the freeze-dried broccoli sprouts was performed in a single procedure. The extraction of phytochemicals and further desulfation of glucosinolates, was performed as described by Villarreal-García et al. [11] with modifications in the extraction solvent selection. Briefly, 10 mL of ethanol/water (70:30, *v/v*) previously heated for 10 min at 70 °C in a reciprocating water bath (VWR, Radnor, PA, USA), were added to broccoli sprouts powder (0.2 g) followed by the addition of 50 µL of a 3 mM solution of sinigrin as internal standard (I.S). Samples were incubated at 70 °C for 30 min and vortexed at 0, 10 and 20 min to ensure myrosinase inactivation. After removal from the water bath, extracts were left to cool at room temperature and centrifuged (18,000× *g*, 10 min, 4 °C). The clarified extract (supernatant) was recovered for glucosinolate, phenolic and carotenoid/chlorophyll analysis.

#### 3.4.2. Analysis of Glucosinolates

##### Desulfation of Glucosinolates

Immediately after the extraction of phytochemicals, glucosinolates were desulfated and purified using disposable polypropylene columns (Thermo Fisher Scientific, Waltham, MA, USA). To prepare the columns, 0.5 mL of water was added, followed by 0.5 mL of previously prepared Sephadex A-25 and an additional 0.5 mL of water. Clarified ethanolic extract supernatant (3 mL) was added into a prepared column and allowed to drip through slowly. Columns were washed with 2 × 0.5 mL of water followed by 2 × 0.5 mL of 0.02 M sodium acetate. Purified sulfatase (75 μL) was added to each sample and left at room temperature overnight (12 h). Desulfoglucosinolates were eluted with a total of 1.25 mL of water (0.5 mL + 0.5 mL + 0.25 mL).

##### Identification and Quantification of Desulfoglucosinolates by HPLC-DAD and HPLC-ESI-MS^n^

Determination of desulfoglucosinolates was performed as described by Moreira-Rodríguez et al. [27] with the only modification being a slightly longer run time. Briefly, the HPLC system used was composed of a quaternary pump, an autosampler, and a diode array detector (DAD) (1260 Infinity, Agilent Technologies, Santa Clara, CA, USA). Desulfoglucosinolates were separated on a 4.6 mm × 250 mm, 5 μm, C18 reverse phase column (Luna, Phenomenex, Torrace, CA, USA). Water (phase A) and acetonitrile (phase B) were used as mobile phases with a flow rate of 1.5 mL/min and a gradient of 0/100, 28/80, 35/100 (min/% phase A) with an injection volume of 20 µL. Desulfoglucosinolates were detected at 227 nm. Chromatographic data was processed with OpenLAB CDS ChemStation software (Agilent Technologies, Santa Clara, CA, USA).

For the HPLC-ESI-MS^n^ analysis, a MS Finnigan LCQ Deca XP Max, Ion trap mass spectrometer coupled at the exit of the DAD and equipped with a Z-spray ESI source, and run by Xcalibur version 1.3 software (Thermo Finnigan-Surveyor San José, CA, USA) was used. Separations were conducted using the Phenomenex (Torrance, CA, USA) Synergi™ 4 µm Hydro-RP 80 Å (2 mm × 150 mm) with a C18 guard column. The gradient of the solvent system used was 0/99, 16/80, 18/10 (min/% phase A) and a flow rate of 350 µL/min from the DAD eluent was directed to the ESI interface using a flow-splitter. Nitrogen was used as a desolvation gas at 275 °C and a flow rate of 60 L/h, and helium was used as damping gas. ESI was performed in the negative ion mode using the following conditions: sheath gas (N_2_), 60 arbitrary units; spray voltage, 5 kV; capillary temperature, 285 °C; capillary voltage, 48.5 V; and tube lens offset, 30 V.

Individual glucosinolates were identified on the basis of retention time, UV spectra, and their mass-to-charge (*m*/*z*) ratio as compared with authentic standards and previous literature data [9,10,11,57,58,59]. For the quantification of glucosinolates, a standard curve of desulfoglucoraphanin was prepared in the range of 0–700 μM. The concentration of total and individual glucosinolates was expressed as mmol of desulfoglucoraphanin equivalents per g of broccoli sprouts dry weight (DW).

#### 3.4.3. Analysis of Phenolic Compounds

##### Identification and Quantification of Phenolic Compounds by HPLC-DAD and HPLC-ESI-MS^n^

The identification and quantification of individual phenolic compounds were performed according to Torres-Contreras et al. [60] with slight modifications described by Moreira-Rodríguez et al. [27]. Briefly, 10 µL of clarified ethanolic extracts, previously filtered using 0.45 µm nylon membranes (VWR, Radnor, PA, USA), were injected in the HPLC-DAD system (1260 Infinity, Agilent Technologies, Santa Clara, CA, USA). Compounds were separated on a 4.6 mm × 250 mm, 5 µm particle size, C18 reverse phase column (Luna, Phenomenex, Torrance, CA, USA). Mobile phases consisted of water (phase A) and methanol:water (60:40, *v*/*v*, phase B) both adjusted at pH 2.4 with orthophosphoric acid. The gradient solvent system was 0/100, 3/70, 8/50, 35/30, 40/20, 45/0, 50/0, and 60/100 (min/% phase A) at a constant flow rate of 0.8 mL/min. Phenolic compounds were detected at 280, 320 and 360 nm.

The same HPLC solvent gradient was used for the HPLC-ESI-MS^n^ analysis to obtain mass spectra of compounds. Mobile phases were adjusted to pH 2.4 with formic acid. The flow rate was 200 µL/min. Nitrogen was used as desolvation gas at 275 °C and a flow rate of 60 L/h. Helium was used as the damping gas. ESI was performed in the negative ion mode using the following conditions: sheath gas (N_2_), 60 arbitrary units; spray voltage, 1.5 kV; capillary temperature, 285 °C; capillary voltage, 45.7 V; and tube lens offset, 30 V.

Individual phenolics were identified on the basis of retention time, UV spectra and their mass-to-charge ratio as compared with authentic standards and reported data [9,10,11,61,62,63,64]. To quantify phenolic compounds, standard curves of sinapic acid (0–100 ppm), ferulic acid (0–20 ppm), gallic acid (0–20 ppm) and 3-*O*-CQA (0–20 ppm) were prepared. Thus, the concentration of individual phenolic compounds was expressed as mg of sinapic acid, ferulic acid, gallic acid, or 3-*O*-CQA equivalents per kg of broccoli sprouts DW, as appropriate. Similarly, the concentration of total phenolics (mg/kg DW) was determined as the sum of all individual phenolic compounds.

#### 3.4.4. Analysis of Carotenoids and Chlorophylls

##### Identification and Quantification of Carotenoids and Chlorophylls by HPLC-DAD

Identification and quantification of individual phenolic compounds were performed as described by Sánchez et al. [51]. Briefly, 25 µL of clarified ethanolic extracts, previously filtered using 0.45 µm nylon membranes (VWR, Radnor, PA, USA), were injected in the HPLC-DAD system (1260 Infinity, Agilent Technologies, Santa Clara, CA, USA). Carotenoids and chlorophylls were separated on a C30 reverse phase column (4.6 mm × 150 mm, 3 µm particle size) (YMC, Wilmington, NC, USA), coupled to a corresponding C30 guard cartridge. The temperature of the vial chamber was 4 °C, and column temperature was 30 °C. The mobile phase consisted of 50% methanol, 45% MTBE, and 5% water. The system was isocratic. Total elution time was 35 min at a constant flow rate of 0.5 mL/min. Carotenoids and chlorophylls were detected at 450 nm, and identified by comparing their retention times and absorption spectra with reference standards or previous reports [18,49,50,51]. Xanthophylls were quantified using a calibration curve of lutein standard (0–12 ppm), and expressed as mg of lutein equivalents per kg of broccoli sprouts DW. For the quantification of chlorophylls, a standard curve of chlorophyll *b* was prepared in the range of 10–175 ppm. The concentration of total and individual chlorophylls was expressed as mg of chlorophyll *b* equivalents per kg of broccoli sprouts DW.

### 3.5. Statistical Analysis

Statistical analyses of chemical analyses were performed using three treatment repetitions. Data represent the mean values of samples and their standard error. Analyses of variance (ANOVA) were conducted using JMP software version 12.0 (SAS Institute Inc., Cary, NC, USA) and mean separations performed using the LSD test (*p* < 0.05).

## 5. Conclusions

The increasing recognition of broccoli sprouts as a ready-to-eat source of phytochemicals with anti-cancer, anti-degenerative, and antioxidant properties has led scientific efforts towards the search for improvement in their phytochemical content. Results discussed herein showed that simple pre-harvest treatments such as UV radiation, applied alone or in combination with exogenous MJ, can be used as an effective emerging technology that allows the accumulation of specific phytochemicals in broccoli sprouts. Furthermore, results demonstrated that the profile of glucosinolates accumulated in stressed broccoli sprouts could be tailored towards the over-production of most indole glucosinolates by applying 25 µM MJ alone or preferably in combination with a 120 min exposure to UVA or UVB radiation (9.47 and 7.16 W/m^2^, respectively) 24 h prior harvest. Specifically, a synergistic effect in the accumulation of NGBS was achieved by combining UV and MJ stresses. On the other hand, the production of aliphatic or specific indole glucosinolates can be triggered by UVB supplementation alone. MJ treatments may be applied if an increase in gallic acid, its derivative GAH II, specific sinapic acid derivatives (e.g., 5-SQA) and ferulic acid derivatives (e.g., 1,2-diFG) is desired. However, such increases would be at the expense of the following compounds: GAH I, GTA, diGH, 3-*O*-H-K, 1-*O*-S-β-d-g, sinapoyl malate, sinapic acid, K-3-*O*-S-so-7-*O*-g, 1,2-diSG, 1-S-2-FG, the majoritarian isomer of 1,2,2-triSG and 1,2-diS-1-FG; as they were significantly reduced after treatments with MJ. Application of UVA alone may be recommended to accumulate GAH I, 1-*O*-S-β-d-g, sinapic acid, gallic acid, K-3-*O*-S-so-7-*O*-g, 1-S-2-FG and the second isomer of 1,2,2-triSG. Finally, a single 120 min exposure to UVA radiation should be applied to increase xanthophyll and chlorophyll content in broccoli sprouts.

The results presented herein suggest a complex cross-talk between UV radiation and MJ stresses acting on the metabolism of phytochemicals by redirecting the carbon flux to the biosynthesis of specific glucosinolates, phenolics, carotenoids, and chlorophylls depending on the type of stress applied. Finally, UV- and/or MJ-treated broccoli sprouts with increased concentrations of phytochemicals could be used for fresh consumption or used as a starting material for the extraction of high-value antioxidant, UV-absorbing, photo-protective, and anti-carcinogenic compounds with potential applications in the pharmaceutical, nutraceutical, cosmetic, skin care, and dietary supplements industries.

## Figures and Tables

**Table 1:** Identification of individual desulfoglucosinolates (dsg) in broccoli sprouts. Identification was obtained by HPLC-DAD and HPLC-Electrospray Ionization (ESI)-Sequential Mass Spectrometry (MS^n^).

Peak Number (Retention Time, min)	λ_max_ (nm)	Identification	[M − H]^−^ (*m/z*)	MS^2^ (*m/z*) ^a^
1 (5.3)	222	Glucoiberin-dsg	342	**179**, 131
2 (5.8)	224	Progoitrin-dsg	308	**145**, 129, 79
3 (6.6)	222	Glucoraphanin-dsg	356	**193**
4 (13.6)	221, 266	4-hydroxy-glucobrassicin-dsg	383	**221**, 203, 153
5 (17.9)	210	Glucoerucin-dsg	340	**177**, 160, 129, 113
6 (20.6)	220, 280	Glucobrassicin-dsg	367	**204**, 187, 155, 129
7 (24.3)	220, 268	4-methoxy-glucobrassicin-dsg	397	**234**, 204, 154, 139
8 (30.3)	222, 290	Neoglucobrassicion-dsg	397	**234**, 204, 154, 129

^a^ Major fragment ions are highlighted in bold.

**Table 2:** Concentration of individual and total glucosinolates in broccoli sprouts treated with UVA or UVB light, methyl jasmonate or a combination of stresses.

**Treatment ^4^**	**Glucosinolate Concentration (mmol/kg DW) ^1,2,3^**									
	**GIB**		**PRO**		**GRA**		**4-HGBS**		**GER**	
Control	2.8 ± 0.4	b	0.7 ± 0.1	a	13.2 ± 1.4	bc	5.4 ± 0.3	b	4.1 ± 0.4	b
UVA	3.2 ± 0.3	b	0.6 ± 0.1	a	13.8 ± 0.9	bc	6.7 ± 1.1	ab	3.0 ± 0.3	c
UVB	5.1 ± 1.2	a	0.8 ± 0.1	a	23.6 ± 2.1	a	7.1 ± 0.4	a	6.6 ± 0.4	a
MJ	2.6 ± 0.2	b	0.4 ± 0.0	b	13.4 ± 0.6	bc	0.5 ± 0.1	c	0.3 ± 0.1	d
UVA + MJ	2.8 ± 0.1	b	0.6 ± 0.1	ab	11.7 ± 1.0	c	1.6 ± 0.2	c	0.8 ± 0.1	d
UVB + MJ	3.2 ± 0.3	b	0.8 ± 0.1	a	15.5 ± 0.8	b	1.3 ± 0.2	c	0.9 ± 0.2	d
**Treatment ^4^**	**Glucosinolate Concentration (mmol/kg DW) ^1,2,3^**									
	**GBS**		**4-MGBS**		**NGBS**		**TOTAL**			
Control	10.3 ± 1.6	d	4.6 ± 0.3	d	15.1 ± 1.4	c	56 ± 5	d		
UVA	11.5 ± 0.9	cd	3.9 ± 0.2	d	10.4 ± 1.5	c	53 ± 1	d		
UVB	13.4 ± 0.9	bc	12.7 ± 0.5	a	12.3 ± 2.1	c	82 ± 5	c		
MJ	15.1 ± 0.8	ab	6.4 ± 0.3	c	72.1 ± 3.5	b	111 ± 5	b		
UVA + MJ	17.7 ± 0.3	a	10.6 ± 0.6	b	96.4 ± 1.5	a	142 ± 3	a		
UVB + MJ	15.2 ± 0.5	ab	9.4 ± 0.6	b	92.8 ± 6.1	a	139 ± 6	a		

^1^ Concentrations are reported as desulfoglucoraphanin equivalents. All compounds were quantified at 227 nm. ^2^ Values represent the mean of three replicates ± standard error of the mean. ^3^ Different letters in the same column indicate statistical difference in the concentration of each compound between treatments using the LSD test (*p* < 0.05). ^4^ For treatments with MJ, 65 mL of a 25 µM MJ solution were applied to broccoli sprouts every 12 h by exogenous spraying from sowing day until the end of the experiment (8th day after sowing). For treatments with UV lights, broccoli sprouts were exposed for 120 min to UVA (9.47 W/m^2^) or UVB (7.16 W/m^2^) light on the 7th day after sowing. Harvest of UVA- or UVB-treated sprouts (with or without MJ) was performed 24 h after the UV treatment. For control and MJ (no UV)-treated sprouts, harvest occurred at the 7th day + 24 h after sowing, without any UV treatment. Abbreviations: Dry weight (DW); Methyl Jasmonate (MJ); Ultraviolet (UV); Glucoiberin (GIB); Progoitrin (PRO); Glucoraphanin (GRA); 4-hydroxy-glucobrassicin (4-HGBS); Glucoerucin (GER); Glucobrassicin (GBS); 4-methoxy-glucobrassicin (4-MGBS), Neoglucobrassicin (NGBS).

**Table 3:** Identification of individual phenolic compounds in broccoli sprouts. Identification was obtained by HPLC-DAD and HPLC-ESI-MS^n^.

Peak Number (Retention Time, min)	λ_max_ (nm)	Identification	[M − H]^−^ (*m/z*)	MS^2^ (*m/z*) ^a^
1 (4.2)	262	Gallic acid hexoside I	331	162, **125**
2 (6.9)	210, 300	Gallotannic acid	1700	1530, **1378**, 1225, 1091
3 (10.7)	272	*p*-hydroxybenzoic acid	137	122, **111**, 107
4 (11.8)	218, 280	Gallic acid hexoside II	331	**162**, 125
5 (12.2)	218sh, 326sh	4-*O*-caffeoylquinic acid	353	**191**, 179, **173**
6 (12.7)	220, 268	digalloyl hexoside	483	337, **169**
7 (13.6)	222, 265, 330	3-*O*-hexoside kaempferol	447	**285**
8 (14.6)	220, 268	Gallic acid derivative	-	-
9 (15.3)	240sh, 328	1-*O*-sinapoyl-β-d-glucose	385	**223**, 205, **173**, 145
10 (16.2)	240sh, 330	Sinapoyl malate	339	**205.6**, **173**, 147, 132
11 (17.2)	228, 330	1,2-diferuloylgentiobiose	693	499, **175**
12 (22.5)	220, 268	5-sinapoylquinic acid	397	**222**, 191
13 (27. 1)	235, 324	Sinapic acid	223	**179**, **163**, 135, 119
14 (29.3)	221, 290	Gallic acid	169	167, 141, **137**, 125, 81
15 (36.2)	238sh, 270, 330	Kaempferol 3-*O*-sinapoyl-sophoroside 7-*O*-glucoside	977	771, **609**, 429, **285**
16 (37.6)	240sh, 268, 332	1,2-disinapoylgentiobiose	753	529, **223**
17 (39.9)	240sh, 330	1-sinapoyl-2′-ferulolylgentiobiose	723	449, **223**
18 (42.4)	240sh, 328	1,2,2′-trisinapoylgentiobiose ^b^	959	735, **223**
19 (43.2)	240sh, 331	1,2-disinapoyl-1′-ferulolylgentiobiose	929	705, **223**
20 (43.9)	220, 238, 328	1,2-disinapoyl-2′-ferulolylgentiobiose	929	705, **223**
21 (46.6)	242, 326	1-sinapoyl-2,2′-diferuloylgentiobiose	899	705, **223**
22 (51.2)	238sh, 330	1,2,2′-trisinapoylgentiobiose ^b^	959	735, **223**

Abbreviations: Shoulder (sh). ^a^ Major fragment ions are highlighted in bold. ^b^ Isomeric compounds.

**Table 4:** Concentration of individual and total phenolic compounds in broccoli sprouts treated with UVA or UVB light, methyl jasmonate or a combination of stresses.

**Treatment ^5^**	**Phenolic Concentration (mg/kg DW) ^1,2,3,4^**															
	**GAH I**		**GTA**		***p*-HBA**		**GAH II**		**4-*O*-CQA**		**diGH**		**3-*O*-H-K**		**GAD**	
Control	522.6 ± 13.0	b	216.6 ± 6.9	a	321.9 ± 25.9	b	330.6 ± 10.4	cd	373.7 ± 58.3	a	310.5 ± 6.0	a	482.2 ± 26.9	a	158.9 ± 14.1	ab
UVA	577.3 ± 17.0	a	218.5 ± 6.5	a	302.3 ± 9.5	bc	386.9 ± 18.6	bc	394.0 ± 61.2	a	321.1 ± 25.8	a	442.3 ± 24.6	a	144.9 ± 7.0	ab
UVB	532.5 ± 23.6	b	224.0 ± 6.8	a	296.7 ± 19.3	bc	446.6 ± 19.6	ab	352.7 ± 43.1	a	283.7 ± 10.3	a	389.3 ± 5.9	b	132.3 ± 4.8	b
MJ	404.6 ± 9.6	c	168.7 ± 4.0	b	266.4 ± 4.7	c	310.8 ± 30.2	d	368.5 ± 15.4	a	214.6 ± 17.5	bc	216.2 ± 3.8	c	165.6 ± 14.0	a
UVA + MJ	388.0 ± 9.3	c	163.5 ± 2.0	b	368.1 ± 5.0	a	468,0 ± 11.3	a	356.5 ± 15.5	a	224.9 ± 14.9	b	206.6 ± 3.0	c	170.2 ± 11.3	a
UVB + MJ	391.5 ± 10.0	c	159.5 ± 3.2	b	301.6 ± 15.9	bc	414.1 ± 32.5	ab	389.6 ± 46.9	a	179.1 ± 5.5	c	211.2 ± 2.7	c	167.0 ± 11.0	a
**Treatment ^5^**	**Phenolic Concentration (mg/kg DW) ^1,2,3,4^**															
	**1-O-S-β-d-g**		**Sinapoyl Malate**		**1,2-diFG**		**5-SQA**		**Sinapic Acid**		**Gallic Acid**		**K-3-O-S-so-7-O-g**		**1,2-diSG**	
Control	559.9 ± 17.4	b	2420.7 ± 82.7	a	468.6 ± 9.0	c	295.6 ± 20.1	c	295.2 ± 34.1	b	201.9 ± 8.3	c	709.6 ± 10.0	b	431.8 ± 7.4	a
UVA	656.3 ± 33.2	a	2579.3 ± 169.6	a	461.9 ± 18.9	cd	212.9 ± 45.5	cd	363.4 ± 15.1	a	317.4 ± 20.4	a	890.0 ± 91.1	a	480.3 ± 43.1	a
UVB	565.8 ± 27.0	b	2605.7 ± 174.6	a	422.6 ± 13.1	d	128.2 ± 16.6	d	265.2 ± 24.6	b	205.7 ± 17.6	c	748.0 ± 25.4	b	445.5 ± 20.5	a
MJ	428.1 ± 10.2	cd	1091.8 ± 73.9	b	617.7 ± 20.6	a	1001.2 ± 65.7	a	154.2 ± 1.5	c	253.9 ± 15.1	b	301.2 ± 14.1	c	325.5 ± 4.8	b
UVA + MJ	454.6 ± 8.1	c	1092.8 ± 14.2	b	589.2 ± 3.8	a	988.5 ± 19.7	a	153.2 ± 1.0	c	237.0 ± 10.7	bc	356.8 ± 27.3	c	332.8 ± 9.4	b
UVB + MJ	383.0 ± 9.8	d	1182.3 ± 13.8	b	539.9 ± 8.8	b	707.8 ± 55.0	b	163.0 ± 2.4	c	228.3 ± 6.0	bc	283.2 ± 3.4	c	326.9 ± 2.8	b
**Treatment ^5^**	**Phenolic Concentration (mg/kg DW) ^1,2,3,4^**															
	**1-S-2-FG**		**1,2,2-triSG ^6^**		**1,2-diS-1-FG**		**1,2-diS-2-FG**		**1-S-2,2-diFG**		**1,2,2-triSG ^6^**		**TOTAL**			
Control	3287.4 ± 55.6	b	9800.6 ± 207.3	a	2501.4 ± 149.7	a	457.1 ± 84.3	a	239.2 ± 7.5	ab	376.5 ± 13.8	c	24,762 ± 477	ab		
UVA	4060.6 ± 424.5	a	11,078.0 ± 1145.9	a	2366.8 ± 199.7	a	286.8 ± 49.7	b	271.9 ± 21.2	a	447.8 ± 42.7	a	27,261 ± 2218	a		
UVB	3440.1 ± 80.9	b	9646.5 ± 381.6	a	2276.4 ± 134.6	a	203.2 ± 7.5	b	250.5 ± 12.7	ab	388.6 ± 11.0	bc	24,250 ± 837	b		
MJ	1269.3 ± 19.4	c	7815.6 ± 173.6	b	1615.4 ± 51.2	b	490.9 ± 20.5	a	186.8 ± 8.8	c	411.9 ± 14.3	abc	18,079 ± 272	c		
UVA + MJ	1295.3 ± 26.9	c	8052.7 ± 144.9	b	1510.9 ± 41.1	b	474.6 ± 26.7	a	178.5 ± 9.9	c	410.9 ± 7.5	abc	18,474 ± 266	c		
UVB + MJ	1210.5 ± 21.5	c	7791.3 ± 243.1	b	1535.8 ± 20.2	b	478.8 ± 16.9	a	217.3 ± 21.2	bc	444.6 ± 10.8	ab	17,706 ± 302	c		

^1^ Concentrations are reported as gallic acid equivalents for GAH I, GTA, *p*-HBA, GAH II, diGH, GAD and gallic acid; as 3-*O*-CQA equivalents for 4-*O*-CQA; as ferulic acid equivalents for 1,2-diFG; and as sinapic acid equivalents for 3-*O*-H-K, 1-*O*-S-β-d-g, sinapoyl malate, 5-SQA, sinapic acid, K-3-*O*-S-so-7-*O*-g, 1,2-diSG, 1-S-2-FG, 1,2,2-triSG, 1,2-diS-1-FG, 1,2-diS-2-FG and 1-S-2-diFG. ^2^ Compounds quantified at 280 nm (GAH I, GTA, *p*-HBA, GAH II, diGH, GAD and gallic acid) and at 320 nm (4-*O*-CQA, 1,2-diFG, 3-*O*-H-K, 1-*O*-S-β-d-g, sinapoyl malate, 5-SQA, sinapic acid, K-3-*O*-S-so-7-*O*-g, 1,2-diSG, 1-S-2-FG, 1,2,2-triSG, 1,2-diS-1-FG, 1,2-diS-2-FG and 1-S-2-diFG). ^3^ Values represent the mean of three replicates ± standard error of the mean. ^4^ Different letters in the same column indicate statistical difference in the concentration of each compound between treatments using the LSD test (*p* < 0.05). ^5^ For treatments with MJ, 65 mL of a 25 μM MJ solution were applied to broccoli sprouts every 12 h by exogenous spraying from sowing day until the end of the experiment (8th day after sowing). For treatments with UV lights, broccoli sprouts were exposed for 120 min to UVA (9.47 W/m^2^) or UVB (7.16 W/m^2^) light on the 7th day after sowing. Harvest of UVA- or UVB-treated sprouts (with or without MJ) was performed 24 h after the UV treatment. For control and MJ (no UV)-treated sprouts, harvest occurred at the 7th day + 24 h after sowing, without any UV treatment. ^6^ Isomeric compounds. Abbreviations: Dry weight (DW); Methyl Jasmonate (MJ); Ultraviolet (UV); gallic acid hexoside I (GAH I); gallotannic acid (GTA); *p*-hydroxybenzoic acid (*p*-HBA); gallic acid hexoside II (GAH II); 4-*O*-caffeoylquinic acid (4-*O*-CQA); digalloyl hexoside (diGH); 3-*O*-hexoside kaempferol (3-*O*-H-K); gallic acid derivative (GAD); 1-*O*-sinapoyl-β-d-glucose (1-*O*-S-β-d-g); 1,2-diferulolylgentiobiose (1,2-diFG); 5-sinapoylquinic acid (5-SQA); kaempferol 3-*O*-sinapoyl-sophoroside 7-*O*-glucoside (K-3-*O*-S-so-7-*O*-g); 1,2-disinapoylgentiobiose (1,2-diSG); 1-sinapoyl-2′-ferulolylgentiobiose (1-S-2-FG); 1,2,2′-trisinapoylgentiobiose (1,2,2-triSG); 1,2-disinapoyl-1′-ferulolylgentiobiose (1,2-diS-1-FG); 1,2-disinapoyl-2-ferulolylgentiobiose (1,2-diS-2-FG); 1-sinapoyl-2,2′-diferulolylgentiobiose (1-S-2,2-diFG).

**Table 5:** Identification of individual carotenoids and chlorophylls in broccoli sprouts.

Peak Number (Retention Time, min)	λ_max_ (nm)	Tentative Identification	Method of Identification ^a^
1 (5.3)	422sh, 445, 474	Lutein	A, B, C
2 (6.1)	461	Chlorophyll *b*	A, B, C
3 (6.9)	417sh, 441, 470	Neoxanthin	A, B, C
4 (7.6)	335sh, 381sh, 413sh, 432	Chlorophyll *a*	A, B, C

Abbreviations: shoulder (sh). ^a^ Identification of each peak was performed by (**A**) Comparison with the retention time and wavelengths of maximum absorption in the UV/Vis spectra of commercial standards; (**B**) identification by spectral interpretation of the wavelengths of maximum absorption in the UV/Vis spectra and comparison with wavelengths of maximum absorption reported in the literature [17,18,47,48,49,50]; and (**C**) identification by the order of chromatographic elution reported in the literature [17,18,49,50,51].

**Table 6:** Concentration of carotenoids and chlorophylls in broccoli sprouts treated with UVA or UVB light, methyl jasmonate, or a combination of stresses.

Treatment ^4^	Carotenoid/Chlorophyll Concentration (mg/kg DW) ^1,2,3^									
	Lutein		Chlorophyll *b*		Neoxanthin		Chlorophyll *a*		TOTAL	
Control	472.2 ± 22.3	b	6615.2 ± 453.1	b	116.7 ± 7.5	b	1326.6 ± 103.9	b	8531 ± 559	b
UVA	577.9 ± 39.4	a	8647.9 ± 803.1	a	155.8 ± 10.2	a	2216.1 ± 308.6	a	11,598 ± 1159	a
UVB	552.1 ± 28.7	a	7547.1 ± 386.2	ab	159.5 ± 6.8	a	1814.6 ± 157.6	a	10,073 ± 569	ab
MJ	228.8 ± 8.1	c	3174.8 ± 183.9	c	68.5 ± 5.1	c	744.1 ± 35.0	c	4216 ± 218	c
UVA + MJ	235.9 ± 6.5	c	2947.4 ± 83.8	c	69.2 ± 1.6	c	548.0 ± 7.3	c	3800 ± 93	c
UVB + MJ	210.3 ± 4.8	c	2259.6 ± 71.1	c	58.6 ± 0.6	c	457.8 ± 21.2	c	2986 ± 93	c

^1^ Concentrations of lutein and neoxanthin are reported as lutein equivalents. Chlorophylls are reported as chlorophyll *b* equivalents. All compounds were quantified at 450 nm. ^2^ Values represent the mean of three replicates ± standard error of the mean. ^3^ Different letters in the same column indicate statistical difference in the concentration of each compound between treatments using the LSD test (*p* < 0.05). ^4^ For treatments with MJ, 65 mL of a 25 µM MJ solution were applied to broccoli sprouts every 12 h by exogenous spraying from sowing day until the end of the experiment (8th day after sowing). For treatments with UV lights, broccoli sprouts were exposed for 120 min to UVA (9.47 W/m^2^) or UVB (7.16 W/m^2^) light on the 7th day after sowing. Harvest of UVA- or UVB-treated sprouts (with or without MJ) was performed 24 h after the UV treatment. For control and MJ (no UV)-treated sprouts, harvest occurred at the 7th day + 24 h after sowing, without any UV treatment. Abbreviations: Dry weight (DW); Methyl Jasmonate (MJ); Ultraviolet (UV).

## References

[1] Vale A.P., Santos J., Brito N.V., Fernandes D., Rosa E., Beatriz M., Oliveira P.P. (2015). Evaluating the impact of sprouting conditions on the glucosinolate content of *Brassica oleracea* sprouts. Phytochemistry.

[2] Jeffery E.H., Araya M. (2009). Physiological effects of broccoli consumption. Phytochem. Rev..

[3] Paja̧k P., Socha R., Gałkowska D., Rożnowski J., Fortuna T. (2014). Phenolic profile and antioxidant activity in selected seeds and sprouts. Food Chem..

[4] Fahey J.W., Wehage S.L., Holtzclaw W.D., Kensler T.W., Egner P.A., Shapiro T.A., Talalay P. (2012). Protection of humans by plant glucosinolates: Efficiency of conversion of glucosinolates to isothiocyanates by the gastrointestinal microflora. Cancer Prev. Res..

[5] Dinkova-Kostova A.T., Kostov R.V. (2012). Glucosinolates and isothiocyanates in health and disease. Trends Mol. Med..

[6] Herr I., Büchler M.W. (2010). Dietary constituents of broccoli and other cruciferous vegetables: Implications for prevention and therapy of cancer. Cancer Treat. Rev..

[7] Higdon J., Delage B., Williams D., Dashwood R. (2007). Cruciferous vegetables and human cancer risk: Epidemiologic evidence and mechanistic basis. Pharmacol. Res..

[8] Cartea M.E., Francisco M., Soengas P., Velasco P. (2010). Phenolic Compounds in Brassica Vegetables. Molecules.

[9] Torres-Contreras A.M., Nair V., Cisneros-Zevallos L., Jacobo-Velázquez D.A. (2017). Stability of bioactive compounds in broccoli as affected by cutting styles and storage time. Molecules.

[10] Vallejo F., Tomás-Barberán F., García-Viguera C. (2003). Health-promoting compounds in broccoli as influenced by refrigerated transport and retail sale period. J. Agric. Food Chem..

[11] Villarreal-García D., Nair V., Cisneros-Zevallos L., Jacobo-Velázquez D.A. (2016). Plants as biofactories: Postharvest stress-induced accumulation of phenolic compounds and glucosinolates in broccoli subjected to wounding stress and exogenous phytohormones. Front. Plant Sci..

[12] Jacobo-Velázquez D.A., Cisneros-Zevallos L. (2009). Correlations of antioxidant activity against phenolic content revisited: A new approach in data analysis for food and medicinal plants. J. Food Sci..

[13] Pandey K.B., Rizvi S.I. (2009). Plant polyphenols as dietary antioxidants in human health and disease. Oxid. Med. Cell. Longev..

[14] Torres J.L., Ramos-Romero S., Pérez-Jiménez J., Valenzuela J.C., Vergara-Salinas J.R., Perez-Correa J.R. (2016). Key Aspects of polyphenols and health. Advances in Technologies for Producing Food-Relevant Polyphenols.

[15] Santana-Gálvez J., Cisneros-Zevallos L., Jacobo-Velázquez D.A. (2017). Chlorogenic acid: Recent advances on its dual role as a food additive and a nutraceutical against metabolic syndrome. Molecules.

[16] Clotault J., Peltier D., Berruyer R., Thomas M., Briard M., Geoffriau E. (2008). Expression of carotenoid biosynthesis genes during carrot root development. J. Exp. Bot..

[17] Dos Reis L.C.R., de Oliveira V.R., Hagen M.E.K., Jablonski A., Flôres S.H., de Oliveira Rios A. (2015). Carotenoids, flavonoids, chlorophylls, phenolic compounds and antioxidant activity in fresh and cooked broccoli (*Brassica oleracea* var. *Avenger*) and cauliflower (*Brassica oleracea* var. *Alphina* F1). LWT-Food Sci. Technol..

[18] Villarreal-García D., Alanís-Garza P.A., Cuéllar-Villarreal R., Redondo-Gil M., Mora-Nieves J.L., Jacobo-Velázquez D.A. (2015). Effect of different defrosting methods on the stability of bioactive compounds and consumer acceptability of frozen broccoli. CyTA-J. Food.

[19] Bernal J., Mendiola J.A., Ibáñez E., Cifuentes A. (2011). Advanced analysis of nutraceuticals. J. Pharm. Biomed. Anal..

[20] Niyogi K.K., Wolosiuk R.A., Malkin R., Buchanan B.B., Gruissem W., Jones R.L. (2015). Photosynthesis. Biochemistry and Molecular Biology of Plants.

[21] Ferruzzi M.G., Blakeslee J. (2007). Digestion, absorption, and cancer preventative activity of dietary chlorophyll derivatives. Nutr. Res..

[22] Cisneros-Zevallos L. (2003). The use of controlled postharvest abiotic stresses as a tool for enhancing the nutraceutical content and adding-value. J. Food Sci..

[23] Jacobo-Velázquez D.A., Cisneros-Zevallos L. (2012). An alternative use of horticultural crops: Stressed plants as biofactories of bioactive phenolic compounds. Agriculture.

[24] Jenkins G.I., Brown B.A. (2007). UV-B Perception and signal transduction. Light and Plant Development.

[25] Schreiner M., Mewis I., Huyskens-Keil S., Jansen M.A.K., Zrenner R., Winkler J.B., O’Brien N., Krumbein A. (2012). UV-B-induced secondary plant metabolites-potential benefits for plant and human health. CRC Crit. Rev. Plant. Sci..

[26] Mewis I., Schreiner M., Nguyen C.N., Krumbein A., Ulrichs C., Lohse M., Zrenner R. (2012). UV-B irradiation changes specifically the secondary metabolite profile in broccoli sprouts: Induced signaling overlaps with defense response to biotic stressors. Plant Cell Physiol..

[27] Moreira-Rodríguez M., Nair V., Benavides J., Cisneros-Zevallos L., Jacobo-Velázquez D.A. (2017). UVA, UVB light doses and harvesting time differentially tailor glucosinolate and phenolic profiles in broccoli sprouts. Molecules.

[28] De Geyter N., Gholami A., Goormachtig S., Goossens A. (2012). Transcriptional machineries in jasmonate-elicited plant secondary metabolism. Trends Plant Sci.,.

[29] Pérez-Balibrea S., Moreno D.A., García-Viguera C. (2011). Improving the phytochemical composition of broccoli sprouts by elicitation. Food Chem..

[30] Barrientos Carvacho H., Pérez C., Zúñiga G., Mahn A. (2014). Effect of methyl jasmonate, sodium selenate and chitosan as exogenous elicitors on the phenolic compounds profile of broccoli sprouts. J. Sci. Food Agric..

[31] Mikkelsen M.D., Larsen-Petersen B., Glawischnig E., Bøgh-Jensen A., Andreasson E., Halkier B.A. (2003). Modulation of CYP79 genes and glucosinolate profiles in *Arabidopsis* by defense signaling pathways. Plant Physiol..

[32] Ku K.M., Juvik J.A. (2013). Environmental stress and methyl jasmonate-mediated changes in flavonoid concentrations and antioxidant activity in broccoli florets and kale leaf tissues. Hortic. Sci..

[33] Wiesner M., Hanschen F.S., Schreiner M., Glatt H., Zrenner R. (2013). Induced Production of 1-Methoxy-Indol-3-Ylmethyl Glucosinolate by Jasmonic Acid and Methyl Jasmonate in Sprouts and Leaves of Pak Choi (*Brassica Rapa* ssp. *Chinensis*). Int. J. Mol. Sci..

[34] Skirycz A., Reichelt M., Burow M., Birkemeyer C., Rolcik J., Kopka J., Zanor M.I., Gershenzon J., Strnad M., Szopa J. (2006). DOF transcription factor AtDof1.1 (OBP2) is part of a regulatory network controlling glucosinolate biosynthesis in *Arabidopsis*. Plant J..

[35] Pfalz M., Mikkelsen M.D., Bednarek P., Olsen C.E., Halkier B.A., Kroymann J. (2011). Metabolic engineering in *Nicotiana benthamiana* reveals key enzyme functions in *Arabidopsis* indole glucosinolate modification. Plant Cell.

[36] Pfalz M., Mukhaimar M., Perreau F., Kirk J., Hansen C.I.C., Olsen C.E., Agerbirk N., Kroymann J. (2016). Methyl transfer reactions in glucosinolate biosynthesis mediated by Indole glucosinolate O-methyltransferase 5. Plant Physiol..

[37] Baskar V., Gururani M.A., Yu J.W., Park S.W. (2012). Engineering Glucosinolates in Plants: Current Knowledge and Potential Uses. Appl. Biochem. Biotechnol..

[38] Wang Y., Xu W.J., Yan X.F., Wang Y. (2011). Glucosinolate content and related gene expression in response to enhanced UV-B radiation in *Arabidopsis*. Afr. J. Biotechnol..

[39] Demukra P.V., Abdala G., Baldwin I.T., Ballaré C.L. (2010). Jasmonate-dependent and independent pathways mediate specific effects of solar ultraviolet B radiation on leaf phenolics and antiherbivore defense. Plant Physiol..

[40] Heredia J.B., Cisneros-Zevallos L. (2009). The effect of exogenous ethylene and methyl jasmonate on pal activity, phenolic profiles and antioxidant capacity of carrots (*Daucus carota*) under different wounding intensities. Postharvest Biol. Technol..

[41] Park W.T., Kim Y.B., Seo J.M., Kim S.J., Chung E., Lee J.H., Park S.U. (2013). Accumulation of anthocyanin and associated gene expression in radish sprouts exposed to light and methyl jasmonate. J. Agric. Food Chem..

[42] Verdaguer D., Jansen M.A.K., Llorens L., Morales L.O., Neugart S. (2017). UV-A radiation effects on higher plants: Exploring the known unknown. Plant Sci..

[43] Kim J., Dolan W.L., Anderson N.A., Chapple C. (2015). Indole glucosinolate biosynthesis limits phenylpropanoid accumulation in *Arabidopsis thaliana*. Plant Cell.

[44] Jacobo-Velázquez D.A., González-Agüero M., Cisneros-Zevallos L. (2015). Cross-talk between signaling pathways: The link between plant secondary metabolite production and wounding stress response. Sci. Rep..

[45] Nićiforović N., Abramovič H. (2014). Sinapic acid and its derivatives: Natural sources and bioactivity. Compr. Rev. Food Sci. Food Saf..

[46] Kopsell D.A., Sams C.E. (2013). Increases in shoot tissue pigments, glucosinolates, and mineral elements in sprouting broccoli after exposure to short-duration blue light from light emitting diodes. J. Am. Soc. Hortic. Sci..

[47] Taylor K.L., Brackenridge A.E., Vivier M.A., Oberholster A. (2006). High-performance liquid chromatography profiling of the major carotenoids in *Arabidopsis thaliana* leaf tissue. J. Chromatogr. A.

[48] Lee H.S., Castle W.S., Coates G.A. (2001). High-performance liquid chromatography for the characterization of carotenoids in the new sweet orange (Earlygold) grown in Florida, USA. J. Chromatogr. A.

[49] Becerra-Moreno A., Alanís-Garza P.A., Mora-Nieves J.L., Mora-Mora J.P., Jacobo-Velázquez D.A. (2014). Kale: An excellent source of vitamin C, pro-vitamin A, lutein and glucosinolates. CyTA-J. Food.

[50] Strati I.F., Sinanoglou V.J., Kora L., Miniadis-Meimaroglou S., Oreopoulou V. (2012). Carotenoids from foods of plant, animal and marine origin: An efficient HPLC-DAD separation method. Foods.

[51] Sánchez C., Baranda A.B., De Marañón I.M. (2014). The effect of high pressure and high temperature processing on carotenoids and chlorophylls content in some vegetables. Food Chem..

[52] Koltermann D., Schreiner M., Krumbein A., Mewis I., Ulrichs C., Huyskens-Keil S. UV-B radiation mediated changes of bioactive compounds *Brassica juncea* L.. Proceedings of the 2007 Eco Summit (ES’07).

[53] Wierstra I., Kloppstech K. (2000). Differential effects of methyl jasmonate on the expression of the early light-inducible proteins and other light-regulated genes in barley. Plant Physiol..

[54] Pérez A.G., Sanz C., Richardson D.G., Olías J.M. (1993). Methyl jasmonate vapor promotes *β*-carotene synthesis and chlorophyll degradation in *Golden Delicious* apple peel. J. Plant Growth Regul..

[55] Jung S. (2004). Effect of chlorophyll reduction in *Arabidopsis thaliana* by methyl jasmonate or norflurazon on antioxidant systems. Plant Physiol. Biochem..

[56] Martinez-Villaluenga C., Peñas E., Ciska E., Piskula M.K., Kozlowska H., Vidal-Valverde C., Frias J. (2010). Time dependence of bioactive compounds and antioxidant capacity during germination of different cultivars of broccoli and radish seeds. Food Chem..

[57] Maldini M., Baima S., Morelli G., Scaccini C., Natella F.A. (2012). Liquid Chromatography-Mass Spectrometry approach to study “glucosinoloma” in broccoli sprouts. J. Mass Spectrom..

[58] Barbieri G., Pernice R., Maggio A., De Pascale S., Fogliano V. (2008). Glucosinolates profile of *Brassica Rapa* L. subsp. *Sylvestris* L. *janch*. var. *esculenta* Hort. Food Chem..

[59] Bhandari S.R., Jo J.S., Lee J.G. (2015). Comparison of glucosinolate profiles in different tissues of nine *Brassica* crops. Molecules.

[60] Torres-Contreras A.M., Nair V., Cisneros-Zevallos L., Jacobo-Velázquez D.A. (2014). Plants as biofactories: Stress-induced production of chlorogenic acid isomers in potato tubers as affected by wounding intensity and storage time. Ind. Crops Prod..

[61] Jaiswal A.K., Abu-Ghannam N., Gupta S.A. (2012). Comparative study on the polyphenolic content, antibacterial activity and antioxidant capacity of different solvent extracts of *Brassica oleracea* vegetables. Int. J. Food Sci. Technol..

[62] Ferreres F., Sousa C., Pereira D.M., Valentão P., Taveira M., Martins A., Pereira J.A., Seabra R.M., Andrade P.B. (2009). Screening of antioxidant phenolic compounds produced by in vitro shoots of *Brassica oleracea* L. var. costata DC. Comb. Chem. High Throughput Screen..

[63] Siger A., Czubinski J., Dwiecki K., Kachlicki P., Nogala-Kalucka M. (2013). Identification and antioxidant activity of sinapic acid derivatives in *Brassica napus* L. seed meal extracts. Eur. J. Lipid Sci. Technol..

[64] Sun J., Xiao Z., Lin L., Lester G.E., Wang Q., Harnly J.M., Chen P. (2013). Profiling polyphenols in five *Brassica* species microgreens by UHPLC-PDA-ESI/HRMS. J. Agric. Food Chem..

